# Clinical, pathophysiologic, and genomic analysis of the outcomes of primary head and neck malignancy after pulmonary metastasectomy

**DOI:** 10.1038/s41598-019-49212-y

**Published:** 2019-09-09

**Authors:** Hsueh-Ju Lu, Chih-Cheng Hsieh, Chi-Chun Yeh, Yi-Chen Yeh, Chun-Chi Wu, Feng-Sheng Wang, Jin-Mei Lai, Muh-Hwa Yang, Cheng-Hsu Wang, Chi-Ying F. Huang, Peter Mu-Hsin Chang

**Affiliations:** 1 Division of Medical Oncology, Department of Internal Medicine, Chung Shan Medical University Hospital, Taichung, Taiwan; 2 School of Medicine, Chung Shan Medical University, Taichung, Taiwan; 3 Program in Molecular Medicine, School of Life Sciences, National Yang Ming University, Taipei, Taiwan; 4 Division of Thoracic Surgery, Department of Surgery, Taipei Veterans General Hospital, Taipei, Taiwan; 5 Faculty of Medicine, National Yang Ming University, Taipei, Taiwan; 6 Jin An Clinic, New Taipei City, Taiwan; 7 Department of Pathology and Laboratory Medicine, Taipei Veterans General Hospital, Taipei, Taiwan; 8 Department of Chemical Engineering, National Chung Cheng University, Chiayi, Taiwan; 9 Department of Life Science, Fu Jen Catholic University, New Taipei City, Taiwan; 10 Division of Medical Oncology, Department of Oncology, Taipei Veterans General Hospital, Taipei, Taiwan; 11 Institute of Clinical Medicine, National Yang Ming University, Taipei, Taiwan; 12 Cancer Center, Keelung Chang Gang Memorial Hospital, Keelung, Taiwan; 13 Institute of Biopharmaceutical Sciences, National Yang Ming University, Taipei, Taiwan; 14 Institute of Medicine, Chung Shan Medical University, Taichung, Taiwan; 15 Department of Medical Research, Chung Shan Medical University Hospital, Taichung, Taiwan

**Keywords:** Medical research, Oncology

## Abstract

The median overall survival (OS) of some head and neck malignancies, such as head and neck squamous cell carcinoma (HNSCC), with metastatic lesions was only 12 months. Whether aggressive pulmonary metastasectomy (PM) improves survival is controversial. Patients with primary head and neck malignancy undergoing PM were enrolled. Clinical outcomes were compared among different histological types. Whole-exome sequencing was used for matched pulmonary metastatic samples. The genes where genetic variants have been identified were sent for analysis by DAVID, IPA, and STRING. Forty-nine patients with primary head and neck malignancies were enrolled. Two-year postmetastasectomy survival (PMS) rates of adenoid cystic carcinoma, thyroid carcinoma, nasopharyngeal carcinoma, and HNSCC were 100%, 88.2%, 71.4%, and 59.2%, respectively (*P* = 0.024). In HNSCC, the time to distant metastasis was an independent predictive factor of the efficacy of PM. Several pathways, such as branched-chain amino acid (BCAA) consumption, were significantly associated with the progression of HNSCC [*P* < 0.001, fold enrichment (FE) = 5.45]. Moreover, metabolism-associated signaling pathways also seemed to be involved in cancer metastasis. Histological types and time to distant metastasis were important factors influencing the clinical outcomes of PM. For HNSCC, metabolic-associated signaling pathways were significantly associated with tumor progression and distant metastasis. Future validations are warranted.

## Introduction

Primary head and neck malignancies, including head and neck squamous cell carcinoma (HNSCC), nasopharyngeal carcinoma (NPC), thyroid carcinoma (TC), and adenoid cystic carcinoma (ACC), constitute the fourth most common cancer type globally^1^. At least 10% of patients with primary head and neck malignancy progressed to pulmonary metastasis, which is the most common distant metastatic site^2–6^. Because of the different histological types, distant metastasis has been considered incurable with poor prognosis; the median overall survival (OS) was only 10 to 12 months for some patients, such as those with HNSCC^7,8^. Therefore, whether aggressive pulmonary metastasectomy (PM) is indicated for all primary head and neck malignancies is controversial.

In previous studies, PM has been discussed in depth, but no definite conclusion has been reached. Although some studies showed that PM can prolong OS to up to 24 months^9–12^ and the National Comprehensive Cancer Network (NCCN) guideline also suggested that surgical excision is indicated for selected patients with limited metastases^13^, the critical factor is how to select patients who will benefit from this invasive treatment. Previous studies showed that the disease-free interval, the thoracic lymph node metastases, and the number of pulmonary metastases were prognostic factors for HNSCC patients receiving PM^14^, but there were no clear cutoff markers to select patients to receive PM.

To clarify this point, we first approached patients with different clinical presentations. In addition, pathophysiologic and genomic aspects to determine the differences between patients with or without survival benefit after PM were also analyzed. Patients undergoing PM in our institution were retrospectively enrolled. In our analysis, histological type and time to distant metastasis were important factors influencing the clinical outcomes of PM. After sequencing matched pulmonary metastatic samples of HNSCC, metabolic-associated signaling pathways were significant for patients with HNSCC in terms of tumor progression and distant metastasis.

## Results

### Different prognoses of primary head and neck tumor patients after PM

**Figure 1:**
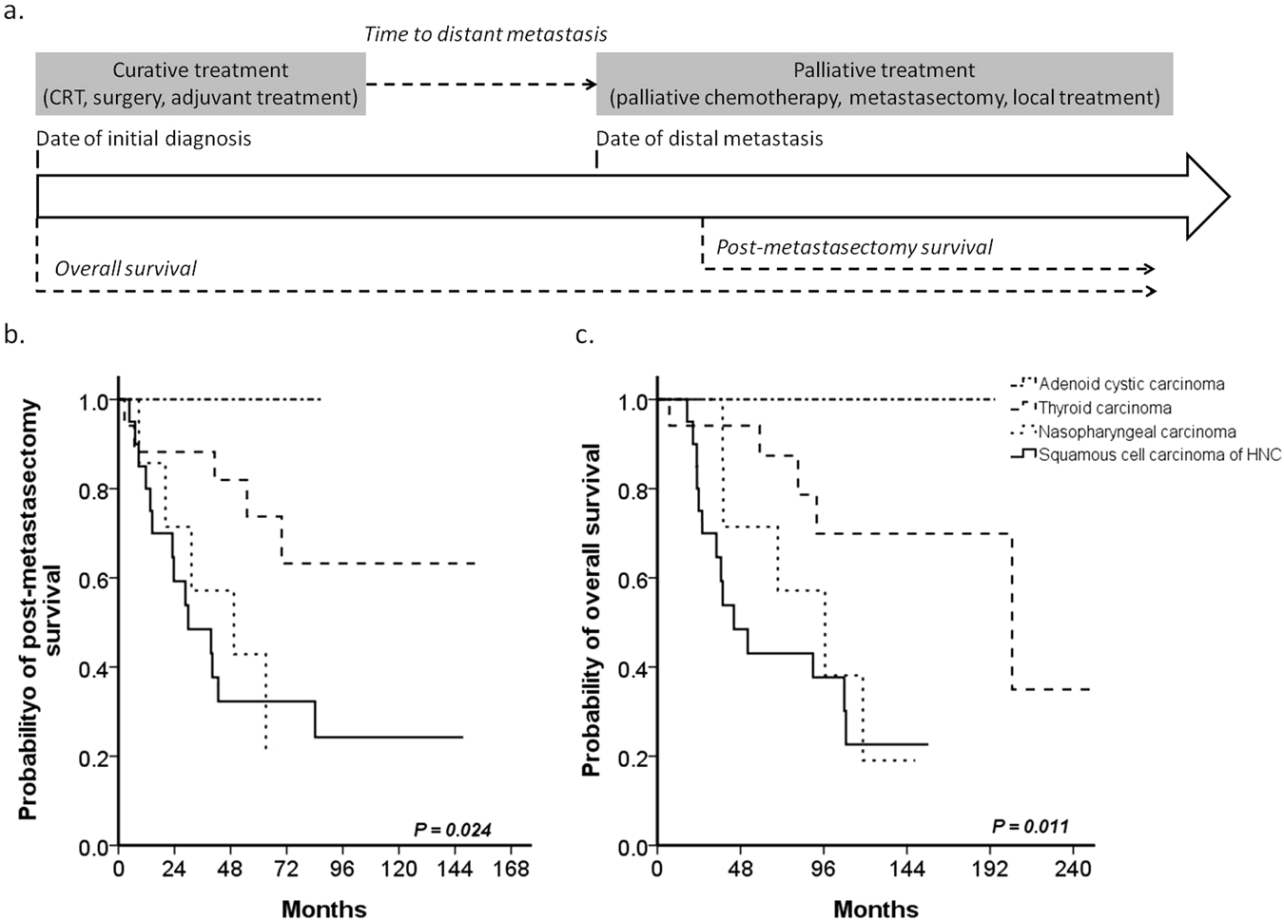
Clinical course and survival outcome of primary head and neck malignancy. (**a**) Clinical course and the definitions of time to distant metastasis, postmetastasectomy survival (PMS), and overall survival (OS). (**b**) Two-year PMS rates of head and neck primary tumors (*P* = 0.024). (**c**) 5-year OSs (*P* = 0.011).

The survival outcomes among the patients with primary head and neck malignancies were significantly different based on histological types. The two-year postmetastasectomy survival (PMS) rates of HNSCC, NPC, TC, and ACC were 59.2%, 71.4%, 88.2%, and 100%, respectively (*P* = 0.024). Most PMS rates of head and neck primary malignancies were longer than 24 months, except for that of HNSCC. Further, the 5-year OS also significantly and was 43.1%, 71.4%, 87.4%, and 100%, respectively (*P* = 0.011) (Fig. 1).

### Chest imaging of pulmonary metastases

**Figure 2:**
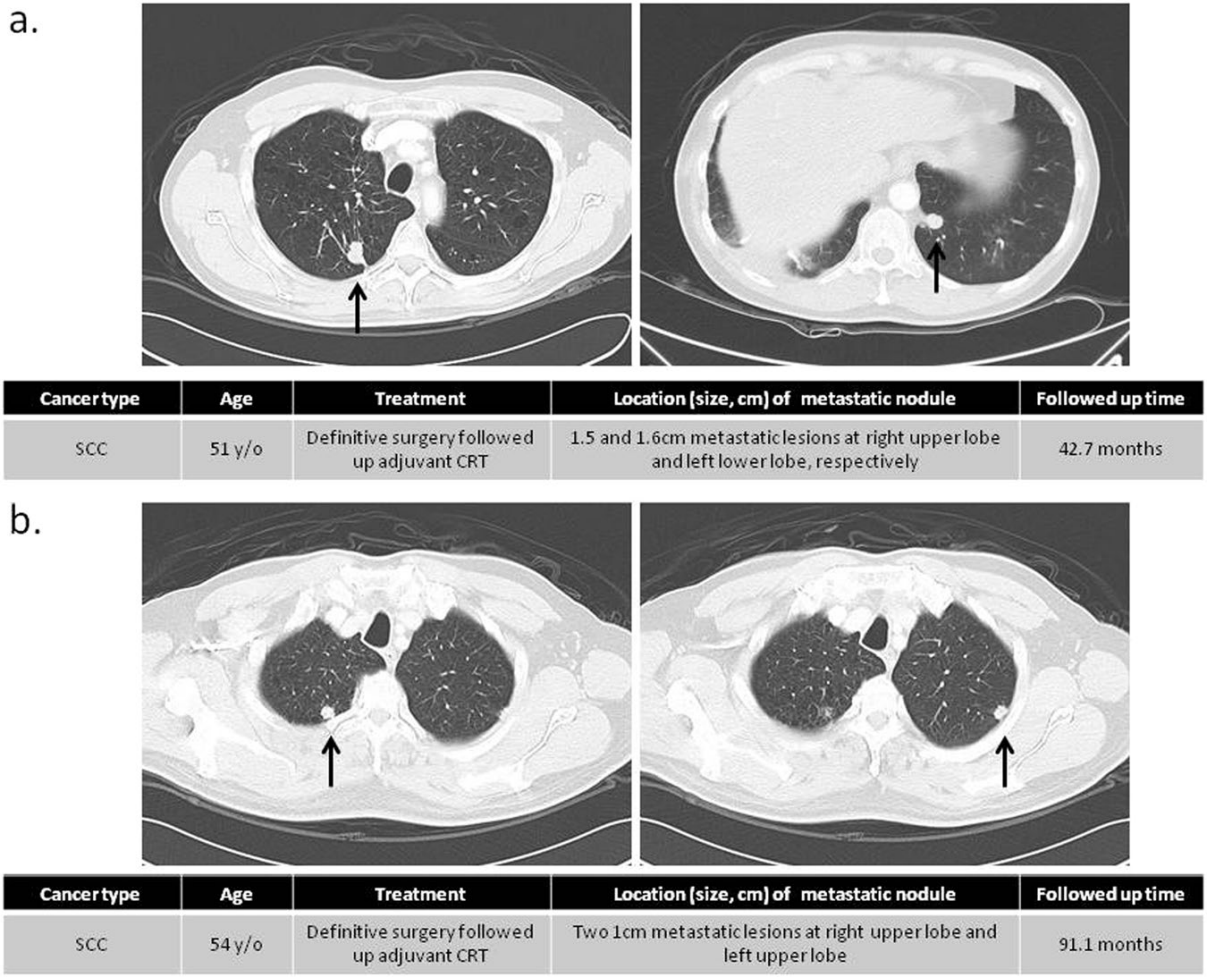
Clinical information and computed tomographies of pulmonary metastases. (**a**) Oligometastases of the bilateral lung were found until month 47.1 following diagnosis, including metastases to the right upper lobe (RUL) and left lower lobe (LLL) of the lung. (**b**) Two subpleural metastatic nodules approximately 1 cm in diameter were found in bilateral apices of the lung 42.4 months after the initial diagnosis. There were no other significant metastatic lesions in the distal lymph nodes, bone, or bilateral adrenal glands.

To exclude possible primary lung tumors, only patients with typical imaging for lung metastasis were included. For example, the clinical information and chest computed tomographies of two patients from our cohort are presented as Fig. 2. The first patient was a 51-year-old male with hypopharyngeal squamous cell carcinoma who was treated with primary wide excision plus lymph node dissection followed by adjuvant chemoradiotherapy. Subsequently, 25.3 months from the initial diagnosis, multiple pulmonary metastases in the right upper lung (RUL), right middle lung, and left lower lung (LLL) were found. Radiofrequency ablation of these lesions was performed several times. Sequential wedge resection for residual RUL and LLL metastatic nodule lesions was performed until 47.1 months after diagnosis (Fig. 2a). The PMS was up to 42.7 months. The other patient was a 54-year-old male with hypopharyngeal squamous cell carcinoma. He also underwent standard treatment after diagnosis. Two subpleural metastatic nodules approximately 1 cm in size were found in the RUL and LUL 42.4 months after the initial diagnosis (Fig. 2b). PM was completed uneventfully in both cases. PMS at the most recent follow-up was 91.1 months. These two cases indicate that PM can be successfully used to treat some patients with metastatic HNSCC and highlights the need for predictive biomarkers to identify these patients.

### Clinical information of patients with HNSCC receiving PM

Forty percent (40.8%, 20/49) of patients with primary head and neck malignancy belonged to the HNSCC group (Table 1). Primary tumor locations of HNSCC were the oropharynx (40%, 8/20), hypopharynx (40%, 8/20), oral cavity (10%, 2/20) and larynx (10%, 2/20). Among the 20 patients in the HNSCC group, three (15%, 3/20) were undergoing palliative therapy because of distant metastases at the initial diagnosis; another 17 patients, with locally advanced status at the initial diagnosis, were treated with curative surgery (65%, 13/20) or curative radiotherapy (20%, 4/20). No patients died during the course of curative treatment. Among the HNSCC patients who received curative therapy, the median time to distant metastasis was 7.5 months (range: 3.6–39.1 months), and 70.1% (12/17) of these patients progressed to distant metastasis within 12 months after completing the initially curative treatments.

**Table 1:** Basic characteristic of head and neck primary cancer patients receiving pulmonary metastasectomy^a^.

Characteristic	HNSCC	NPC	TC	ACC	*P* value
	(N = 20)	(N = 7)	(N = 16)	(N = 3)	
General information					
Age on resected date ≥60 (years)	6 (30.0)	2 (28.6)	5 (31.3)	0 (0.0)	0.733
Male	20 (100.0)	5 (71.4)	8 (50.0)	0 (0.0)	<0.001
Primary tumor location					<0.001
Oral cavity	2 (10.0)	0 (0.0)	0 (0.0)	1 (33.3)	
Nasopharynx	0 (0.0)	7 (100.0)	0 (0.0)	0 (0.0)	
Oropharynx	8 (40.0)	0 (0.0)	0 (0.0)	0 (0.0)	
Hypopharynx	8 (40.0)	0 (0.0)	0 (0.0)	0 (0.0)	
Larynx	2 (10.0)	0 (0.0)	0 (0.0)	0 (0.0)	
Thyroid	0 (0.0)	0 (0.0)	16 (100.0)	0 (0.0)	
Other	0 (0.0)	0 (0.0)	0 (0.0)	2 (66.7)^b^	
Unknown	0 (0.0)	0 (0.0)	0 (0.0)	0 (0.0)	
Initial clinical staging^c^					
T staging ≥ 3	11/16 (68.8)	3/6 (50.0)	6/10 (60.0)	0/1 (0.0)	0.451
N staging positive	15/17 (88.2)	5/6 (88.3)	5/10 (50.0)	0/1 (0.0)	0.072
Initial treatment					<0.001
Curative CRT	0 (0.0)	2 (28.6)	0 (0.0)	0 (0.0)	
Curative radiotherapy alone	0 (0.0)	1 (14.3)	0 (0.0)	0 (0.0)	
IC followed with curative CRT	2 (10.0)	2 (28.6)	0 (0.0)	0 (0.0)	
IC followed with curative radiotherapy	1 (5.0)	0 (0.0)	0 (0.0)	0 (0.0)	
IC followed with curative surgery and adjuvant CRT	1 (5.0)	0 (0.0)	0 (0.0)	0 (0.0)	
Curative surgery	3 (15.0)	0 (0.0)	14 (87.5)	1 (33.3)	
Curative surgery followed with adjuvant CRT	7 (35.0)	0 (0.0)	0 (0.0)	2 (66.7)	
Curative surgery followed with adjuvant radiotherapy	3 (15.0)	0 (0.0)	0 (0.0)	0 (0.0)	
Palliative therapy	3 (15.0)	2 (28.6)	2 (12.5)	0 (0.0)	
Pathologic feature of curative surgery^c^					
Extracapsular spread	8/11 (72.7)	N/A	2/5 (40.0)	0 (0.0)	0.242
Margin ≤ 0.5 cm	8/11 (72.7)	N/A	6/8 (75.0)	1/1 (100.0)	0.834
Lymphovascular invasion	7/12 (58.3)	N/A	4/8 (50.0)	1/1 (100.0)	0.198
Perineural invasion	6/12 (50.0)	N/A	0/7 (0.0)	1/1 (100.0)	0.024
p16^INK4A^	1/4 (25.0)	N/A	N/A	N/A	N/A
Time to distal metastasis after curative treatment ≤ 12 months^d^	12/17 (70.1)	0/5 (0.0)	5/14 (35.7)	0/3 (0.0)	0.009
Pulmonary metastatic number ≥ 2	8 (40.0)	2 (28.6)	10 (62.5)	2 (66.7)	0.342
Bilateral pulmonary metastases	6 (30.0)	0 (0.0)	0 (0.0)	2 (66.7)	0.007
Pathologic features of matched pulmonary metastatic sample					
Tumor size ≥2 cm	11 (55.0)	5 (71.4)	5 (31.3)	2 (66.7)	0.254
Margin ≤0.5 cm	10 (50.0)	5 (71.4)	8 (72.7)	1 (33.3)	0.427
Mediastinum organ involvement	3 (15.0)	1 (14.3)	3 (18.8)	0 (0.0)	0.874
Smoking^c^					
Yes, but quit after diagnosis	8/17 (47.1)	N/A	N/A	N/A	N/A
Yes, and persisted during treatment	2/17 (11.8)	N/A	N/A	N/A	N/A
No	7/17 (41.2)	N/A	N/A	N/A	N/A

^a^Three salivary gland carcinoma patients with histological types of adenocarcinoma, sarcomatoid carcinoma, and undifferentiated carcinoma were excluded.

^b^One from nasal sinus and another from salivary gland.

^c^Denominator was the available patients to be evaluated.

^d^Patients with initial distant metastasis were excluded. The time to distal metastasis data for thyroid carcinoma patients diagnosed outside of our hospital were unavailable.

Abbreviations: HNSCC, head neck squamous cell carcinoma; NPC, nasopharyngeal carcinoma; TC, thyroid carcinoma; ACC, adenoid cystic carcinoma; IC, induction chemotherapy; CRT, chemoradiotherapy.

Because one-half of patients with HNSCC did not have a PMS longer than 24 months, we tried to identify the HNSCC patients who experienced survival benefit after PM. After univariate and multivariate logistic regressions analyses of patients with or without a PMS longer than 24 months, a time to distant metastasis after curative treatment of ≤12 months was a predictive factor of poor prognosis in multivariate analysis with an odds radio (OR) [95% confidence interval (CI)] of −2.5[−1.032- (−0.079)] (*P* = 0.025). In addition, although curative surgery was not an independent prognostic factor, it was still important to predict the efficacy of PM in the univariate analysis (OR [95% CI], 2.183[0.015–1.271], *P* = 0.045) (Table S1).

### Patients with HNSCC with short- and long-term survival after PMS

To elucidate the differences among patients with short- and long-term survival after PMS, whole-exome sequencing was applied for matched pulmonary metastatic samples, and the identified alternative genes were enriched to identify the possible signaling pathways. Patients were divided into short- and long-term PMS HNSCC groups according to whether the PMS was longer than 24 months^9–11^. The median PMS and OS between the short- and long-term survival groups were 13.7 versus 84.2 months (*P* < 0.001) and 23.9 versus 108.8 months (*P* < 0.001), respectively (Fig. S2a).

**Figure 3:**
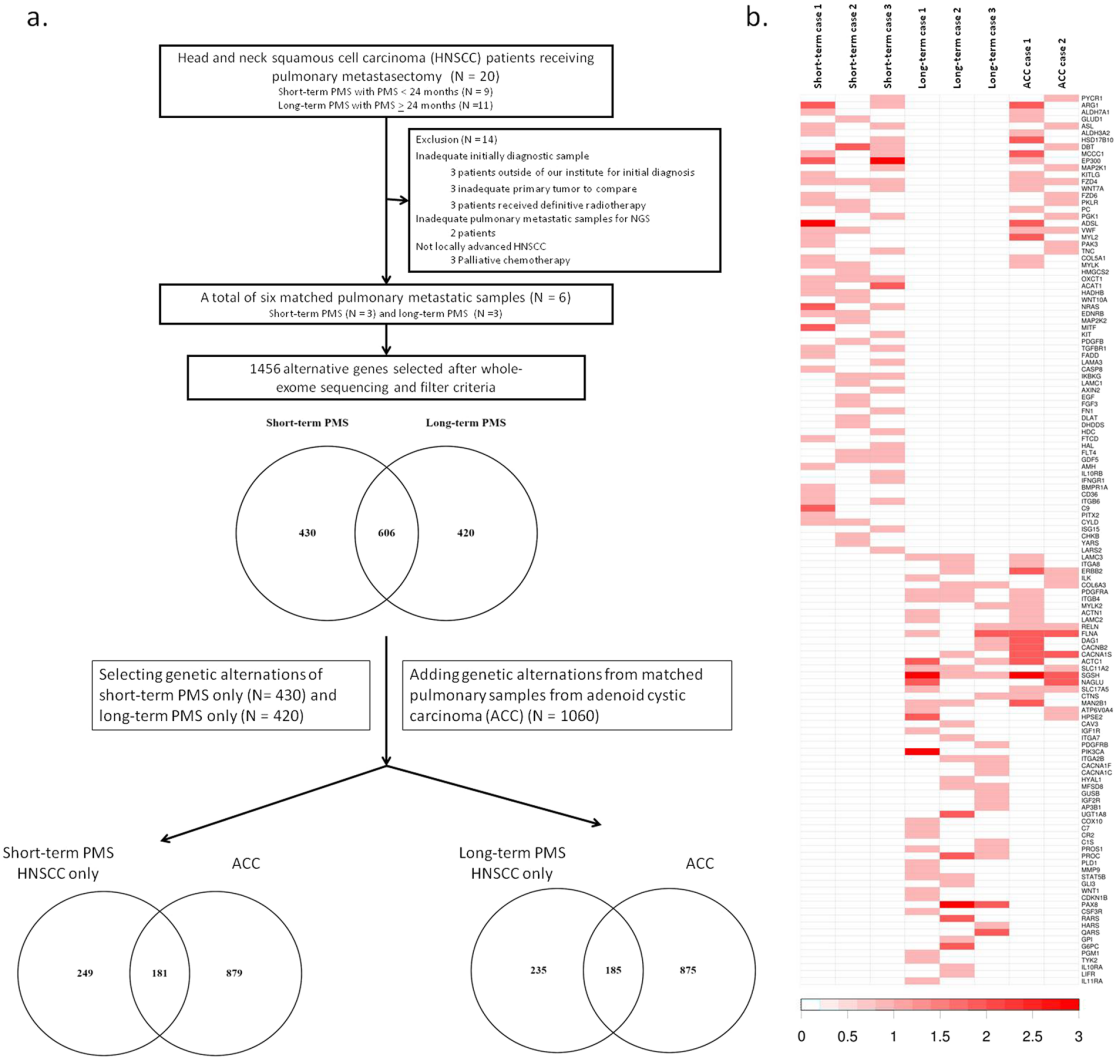
Process and results of whole-exome sequencing. (**a**) After excluding unqualified HNSCC samples, a total of six matched pulmonary metastatic HNSCC samples (three short-term PMS HNSCC and three long-term PMS HNSCC) were sent for whole-exome sequencing. A total of 1,456 alternative genes fulfilled our criteria. These alterative genes were classified into short-term PMS HNSCC only (N = 430), long-term PMS HNSCC only (N = 420), or the intersection between short- and long-term PMS HNSCC (N = 606). Short- or long-term PMS only genes intersected with the identified genes from matched pulmonary metastatic samples of adenoid cystic carcinoma (ACC) (N = 1060). The similar genes of short- or long-term PMS HNSCC and ACC (N = 181, 185, respectively) might indicate cancer metastasis. (**b**) The heatmap of the alternative genes mapped in DAVID. The variant numbers of each gene are represented from white to red (variant numbers to color key, 0 to 0, 1 to 1, 2–3 to 2, 4–10 to 3, 11–25 to 4, > 25 to 5).

Matched pulmonary metastatic samples of these patients were evaluated for whole-exome sequencing. After excluding unqualified samples, six matched pulmonary metastatic samples were selected for further survey (three samples from three corresponding patients with short-term PMS and another three samples from three corresponding patients with long-term PMS) (Figs 3a and S2b). The clinical information of the six selected patients is listed in Table S2.

After sequencing matched samples, the variants, containing both SNP and INDELs, fulfilling our criteria were selected for advanced analysis. The flowchart for selection criteria is shown in Table S3. The finally selected variants, including missense mutations, nonsense mutations, splice sites, and stop-loss mutations, were shown in Table S4. A total of 1,456 alternative genes fulfilled our selection criteria (Fig. 3a,b, Tables S5 and S6). Significantly distinct distributions of the mutant genes of the two groups were evident. In addition, all of the genes were classified into short-term PMS HNSCC only (N = 430), long-term PMS HNSCC only (N = 420), and the intersected genes between the short- and long-term PMS HNSCC (N = 606). The genes presented in the areas without crossover with other groups suggested the unique tumor biological features of the group. Conversely, the intersection area implied potential common features between the two groups. The gene-annotation enrichment analyses of each group are summarized in Table S7.

In the aspect of mutation profiling, branched-chain amino acid (BCAA) consumption (valine/leucine/isoleucine degradation) (*HSD17B10*, *DBT*, *ALDH7A1*, *HMGCS2*, *OXCT1*, *MCCC1*, *ACAT1*, *ALDH3A2*, *HADHB*) [*P* < 0.001, fold enrichment (FE) = 5.45] was the most significant signaling pathway in the short-term PMS HNSCC only group. Other pathways, such as melanogenesis (*P* < 0.001, FE = 3.23) and pathways involved in cancer (*P* < 0.001, FE = 1.95), were also important in this group. However, focal adhesion-associated genes (*CAV3*, *ERBB2*, *ITGB4*, *ACTN1*, *MYLK2*, *FLNA*, *IGF1R*, *LAMC3*, *ITGA8*, *ILK*, *ITGA7*, *COL6A3*, *PDGFRA*, *PDGFRB*, *PIK3CA*, *LAMC2*, *RELN*, *ITGA2B*) played important roles in the long-term PMS HNSCC only group (*P* = 0.003, FE = 2.20) (Table S7).

### The possible genes correlated with cancer metastasis and progression

To identify the possible alternative genes that influenced cancer metastasis, we identified the intersecting alternative genes from matched pulmonary metastatic samples of ACC and those identified from matched pulmonary samples of short- or long-term PMS HNSCC only. As shown in Fig. 1b,c, ACC is a slow-growing but frequently metastatic cancer (the 2-year PMS and 5-year OS rates of our cohort were both 100%)^5,6^. Therefore, the matched pulmonary metastatic samples of ACC might provide more information on cancer metastasis rather than cancer proliferation (Table S8). If we identify the same genes intersected between ACC and HNSCC, these genes might indicate the mechanism of cancer metastasis as well as cancer progression.

Interestingly, a total of 181 alterative genes were intersected between ACC and short-term HNSCC only. The significant pathways were strongly related to metabolic function, such as arginine and proline metabolism (*P* = 0.001, FE = 7.11) and valine/leucine/isoleucine degradation (*P* = 0.005, FE = 7.13) (Tables 2 and S9). Aldehyde dehydrogenase (*ALDH*) gene families, such as *ALDH7A1*, were the most significantly affected downstream genes in these metabolic-associated signaling pathways. In contrast, the intersected genes between ACC and long-term HNSCC were enriched in only focal adhesion (*P* = 0.002, FE = 3.04) and extracellular matrix (ECM)-receptor interaction (*P* = 0.006, FE = 4.24) (Table S9).

**Table 2:** Crossover genes between short-term PMS HNSCC and ACC mapped in DAVID.

Term	Count	Fold-enrichment	P-value	Genes
hsa00330:Arginine and proline metabolism	6	7.11E + 00	1.35E − 03	*PYCR1*, *ARG1*, *ALDH7A1*, *GLUD1*, *ASL*, *ALDH3A2*
hsa00280:Valine, leucine and isoleucine degradation	5	7.13E + 00	4.78E − 03	*HSD17B10*, *DBT*, *ALDH7A1*, *MCCC1*, *ALDH3A2*
hsa04916:Melanogenesis	6	3.80E + 00	1.92E − 02	*EP300*, *MAP2K1*, *KITLG*, *FZD4*, *WNT7A*, *FZD6*
hsa00620:Pyruvate metabolism	4	6.28E + 00	2.44E − 02	*ALDH7A1*, *PKLR*, *ALDH3A2*, *PC*
hsa00010:Glycolysis/Gluconeogenesis	4	4.19E + 00	6.77E − 02	*ALDH7A1*, *PKLR*, *PGK1*, *ALDH3A2*
hsa00250:Alanine, aspartate and glutamate metabolism	3	6.08E + 00	8.48E − 02	*GLUD1*, *ADSL*, *ASL*
hsa04510:Focal adhesion	7	2.19E + 00	9.53E − 02	*VWF*, *MAP2K1*, *MYL2*, *PAK3*, *TNC*, *COL5A1*, *MYLK*

### Ingenuity pathway analysis validation of mapped genes

**Figure 4:**
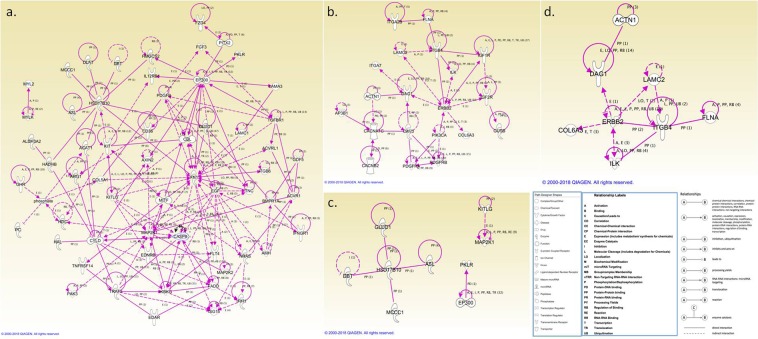
Network analysis mapped genes with validation via ingenuity pathway analysis (IPA). The genes mapped by DAVID were validated with IPA^45^. The selected criteria and gene lists are shown in Table S9. (**a**) The genes from short-term PMS HNSCC only. *HSD17B10* was a hub-node gene. Other genes, such as *FN1*, *EP300*, *CASP8*, *EGF*, and *MAP2K1*, associated with the pathways involved in cancer, were also identified as hub-node genes. (**b**) The genes from long-term PMS HNSCC only. (**c**) The genes from the intersection between short-term PMS HNSCC only and ACC. *HSD17B10* was also a hub-node gene in the crossover mutation between these two groups. (**d**) The genes from the intersection between long-term PMS HNSCC only and ACC.

The genes mapped by DAVID with pathway enrichment *P* values < 0.05 were enrolled for ingenuity pathway analysis (IPA) validation. The selection criteria and number of genes included in the IPA validation are shown in Table S10. As shown in the table, the final numbers of validated genes were 72, 42, 17, and 23 from the short-term PMS HNSCC only, long-term PMS HNSCC only, the intersection between ACC and short-term PMS HNSCC only, and the intersection between ACC and long-term PMS HNSCC only, respectively (Table S10). Most of the top-ranked signaling genes mapping by DAVID were revealed in the IPA. They formed a cluster, and some hub genes were also identified (Fig. 4). For example, among the genes mapped from the short-term HNSCC only group, *HSD17B10*, which was identified as BCAA consumption pathway in DAVID, was a hub-node gene. Others, such as *FN1*, *EP300*, *CASP8*, *EGF* and *MAP2K1*, which were associated with the pathways involved in cancer, were also hub-node genes (Fig. 4a). In addition, *HSD17B10* was also identified as a hub node gene in the intersected mutations between the ACC and short-term PMS HNSCC only groups. The genes that interacted with *HSD17B10*, such as *MCCC1* and *DBT*, were also identified as being involved in BCAA consumption by DAVID (Table 2) (Fig. 4c).

### Gene ontology

The intersecting genes, between the short- or long-term PMS HNSCC only and ACC, were used to further elucidate the cancer metastatic-associated genes. GO of these two intersecting groups were identified by using DAVID and STRING individually. The same GO terms between two databases were listed as Tables S11 and S12. The genes, which appeared in signal pathways and had the same GO terms, were selected as candidate genes. For examples, in the intersecting group between the short-term PMS HNSCC only and ACC, *ALDH7A1*, which presented several times in signal pathways (Table 2) and had the same GO term (GO:0050877), was selected as the candidate gene. Other candidate genes, such as *GLUD1*, *ASL*, and *MCCC1*, were also identified because they presented in signal pathways and GO terms, including glutamine family amino acid metabolic process (GO:0009064) in biologic process, mitochondrion (GO:0005739) in cellular component, and ATP binding (GO:0005524)/adenyl nucleotide binding (GO:0030554) in molecular function, were shown in both databases (Tables S11 and S12).

### *In vivo* and *in vitro* validation

**Figure 5:**
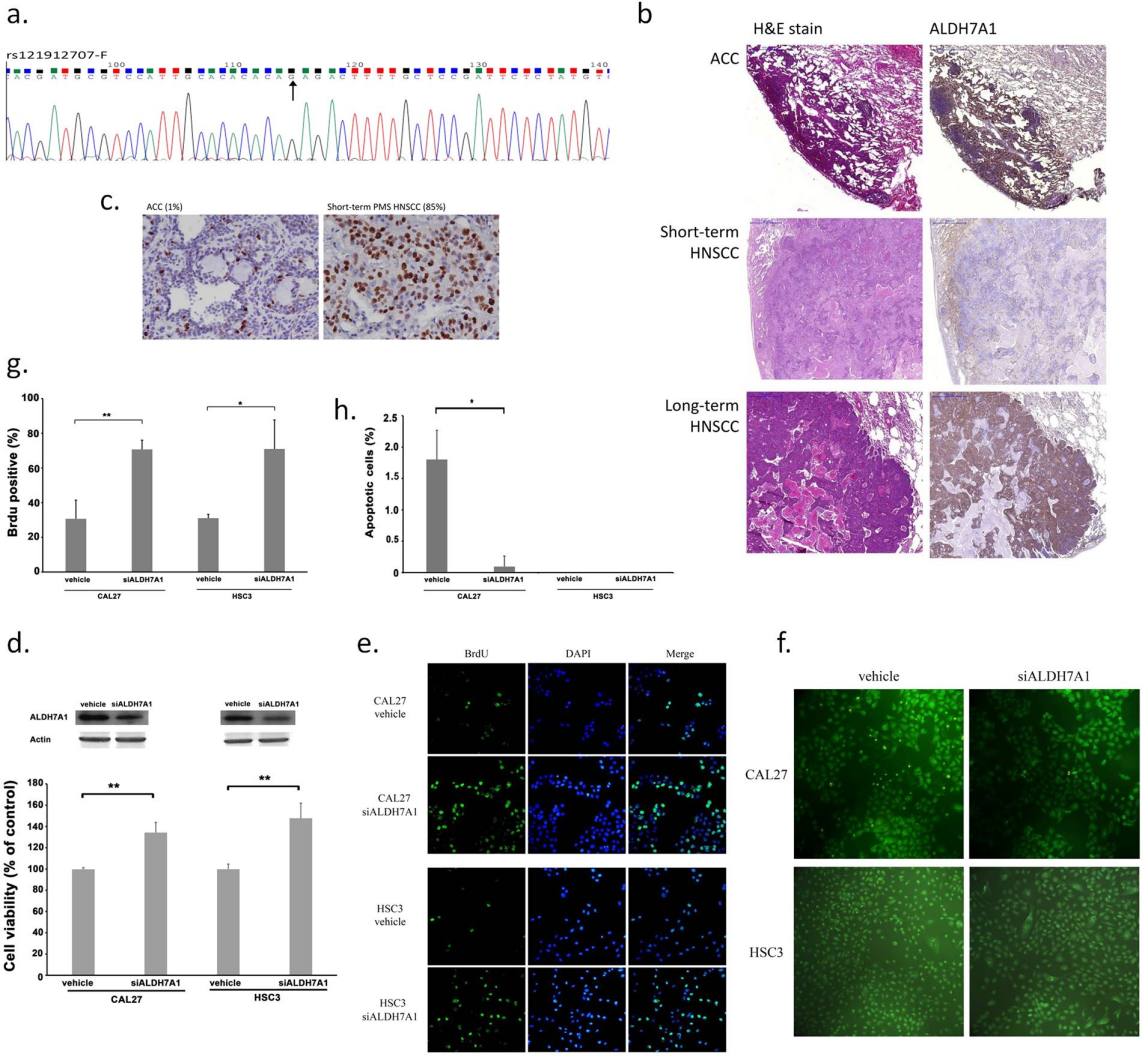
*In vitro* validation of ALDH7A1 mutation. (**a**) Sanger sequencing showed that the point mutation (*ALDH7A1*, missense mutation, c.1168 G > C, rs121912707) was compatible with the results from the high-throughput sequencing (WES). (**b**) Expression level of ALDH7A1 demonstrated by immunohistochemistry (IHC) staining was lower for short-term PMS HNSCC (*ALDH7A1*, missense mutation) than for long-term PMS HNSCC (*ALDH7A1*, wild-type). (**c**) Ki-67 stain of short-term PMS HNSCC was higher than that of ACC (85% vs. 1%) (**d**) After transfecting *ALDH7A1* siRNA in Cal27 and HSC2 cells, the expression of ALDH7A1 was knockdown compared to the expression in the control groups, but cell viability increased. (**e**,**g**) In BrDU assay, knocking down *ALDH7A1* significantly increased the number of synthesized DNA in replicating cell. (**f,h**) In EB/AO assay, apoptotic cells of Cal27 were also decreased after adding si*ALDH7A1*.

We used Sanger sequencing to validate WES’s results and showed the same results (*ALDH7A1*, missense mutation, c.1168 G > C, rs121912707; *FZD4*, missense mutation, c.1250 G > C, rs80358294) (Figs 5a and S3). One patient’s sample, which belonged to short-term HNSCC group and with *ALDH7A1* mutation (rs121912707), was selected to validate the *in silico* result.

Immunohistochemistry (IHC) staining showed that the expression level of ALDH7A1 was lower in the short-term PMS HNSCC patients carrying an *ALDH7A1* mutation (missense, c.1168 G > C, rs121912707) than in the long-term PMS HNSCC patients carrying wild-type *ALDH7A1* (*ALDH7A1* normal expression) (Fig. 5b). Although Ki-67 staining were similar in both short- and long-term PMS HNSCC (data not shown), Ki-67 staining of short-term PMS HNSCC was higher than that of ACC (Fig. 5c). These observations indicate that other factors might influence tumor proliferation rather than metastasis *in vivo*.

For the functional assay, we used two HNSCC cell lines, Cal27 and HSC2, for *in vitro* experiments. Compared to the results in the control group, knocking down *ALDH7A1* decreased ALDH7A1 expression in both Cal27 and HSC2 cell lines but increased the cell proliferation rate (Figs 5d, and S4). *In vitro* validation showed that knockdown ALDH7A1 significantly increased the number of synthesized DNA in replicating cell by BrdU assay (Fig. 5e,g), and apoptotic cells were decreased as detected by EB/AO assay (Fig. 5f,h).

## Discussion

The benefits of PM on primary head and neck malignancies are controversial. The critical factor is large variations in prognosis, and there is no clear cutoff point to select patients who will benefit for the treatment. In this study, a total of 49 patients with primary head and neck malignancy undergoing PM were enrolled. With the exception of patients with HNSCC, the patients with the other head and neck malignancies experienced survival benefits after PM; the 2-year PMS rates for NPC, TC, and ACC were 71.4%, 88.2%, and 100%, respectively. Histological types indeed influenced the outcome of PMS. For HNSCC, time to distant metastasis was a predictive factor for the efficacy of PM. After enriching the results with next-generation sequencing (NGS), metabolic-associated signaling pathways were significant for the short-term PMS HNSCC only group and hinted cancer metastasis. Future validations are warranted *in vitro* and *in vivo*.

In previous studies, PM could improve the OS of some HNSCC patients whose cancer progressed to distant metastasis, but to the criteria used to select suitable patients are controversial^10,12^. The time to distant metastasis had been shown to be a significant prognostic factor for HNSCC^10–12^. In our study, we showed that time to distant metastasis was also a predictive factor for the efficacy of PM^9,11^. It was reasonable that patients with shorter time to distant metastasis had worse PMS because of rapid tumor growth.

Genomic analyses have been widely used to valid HNSCC metastasis in previous publications^15–19^. However, the current study features some novel aspects. First, most researches selected cervical lymph node metastases, which are considered locoregional lesions, to represent the metastatic components of HNSCC^15,17,18^, but only lung or bone metastases are indicated as distant metastases in clinical practice. Because of the different treatment strategies between local recurrence and distant metastatic lesions, we only selected patients with pulmonary metastasis for this analysis. In addition, with the improvement of NGS, we used whole-exome sequencing to identify the novel genomic changes rather than only cancer-related gene changes^17,18^. NGS, including DNA-sequencing technologies and analysis algorithms, was used to maximize the identification of potential pathophysiological signaling pathways^20^. The pathophysiological signaling pathways, which might be unclear, could be identified. These data could provide a more comprehensive picture of tumor physiology.

In addition to a BCAA consumption-associated signaling pathway, the genes associated with short-term HNSCC only were strongly related to pathways involved in cancer based on the DAVID analysis. Some of genes identified from “pathways in cancer” were important in cell proliferation, differentiation, migration, and apoptosis^21–24^. And the hub-node genes identified from the IPA for this group were *FN1*, *EP300*, *CASP8*, *EGF*, and *MAP2K1*. Although metabolic-associated signaling pathways were the most important in the DAVID analysis, *HSD17B10* was the only metabolic-associated hub-node gene. This result might be because less research has focused on the relationship between tumorigenesis and cancer metabolism, so more investigations could be performed in this field. In addition, genes mapped from long-term HNSCC only were correlated with focal adhesion, the top-ranking signaling pathway. Metabolic-associated pathways and focal adhesion/ECM-receptor interaction were mapped from the intersections between short- or long-term PMS and ACC, respectively. Separately, many studies have established the functional complexity of metabolic-associated pathways, including cell proliferation, differentiation, and metastasis^25–27^. In our study, we also found that metabolism-associated pathways, such as BCAA consumption, were significantly associated with patients with short-term PMS. Some of the enzymes involving in BCAA consumption emerged as useful prognostic cancer markers^28,29^. Pre-operation screening, based on metabolic-associated genetic profiles, would be important to select adequate patients receiving PM and large cohort validations were needed. Furthermore, drug developed toward targeting BCAA pathways, especially for high risk patients to prevent distant metastasis and improve clinical outcome may be a novel approach in the future. Focal adhesion and ECM-receptor interactions were also the key determinants regulating cancer migration^30^. In summary, for rapidly progressive tumors, metabolic-associated pathways were important for cancer metastasis; for slow-growing tumors, focal adhesion/ECM-receptor played an important role. The relationship between cancer metabolism and cancer metastasis has been widely discussed^31,32^.

The related studies have shown that the metabolic profiles from liquid samples, such as urine and serum, were significantly different between cancer cells with and without metastasis^31,32^. In addition, to switch their energy source in HNSCC, cancer cells use alternate energy pathways, such as glutaminolysis, which regulates tumorigenesis and cancer stem cell metabolism via aldehyde dehydrogenase (ALDH)^33^. In our study, *ALDH7A1* was selected for validation because recent studies indicated that different ALDH isoforms support increased metastatic capacity in different tumor types^34,35^ and *ALDH7A1* was one of the most significantly affected downstream genes in the metabolic-associated signaling pathways of our results. ALDH also mediated cancer stemness and metastasis in solid tumors^34,36^. Therefore, ALDH is a potential therapeutic target^33,34,36^.

A larger cohort of patients’ samples from corresponding primary cancer, lymph nodes and pulmonary metastasis would be more persuasive. Several reasons prompted us to compared HNSCC and ACC, which are significantly different histological types. First, treatment strategies for these two malignancies are almost the same after distant metastasis. Additionally, the genetic profiles between ACC and HNSCC are more similar than those between ACC and other salivary gland malignancies according to the cohort of Memorial Sloan Kettering Cancer Center (Fig. S5)^37^. Third, ACC is a slow-growing but frequently metastatic cancer. It means the metastatic lesions from ACC might have strong metastatic ability but not cancer proliferation ability. Therefore, we chose the intersected genes between short- or long- term PMS HNSCC only and ACC to separately analyze cancer metastasis and proliferation.

There were some limitations of this study. The first is the small cohort. The number of six matched pulmonary metastatic HNSCC samples was relatively small. This limitation is inevitable for a pilot study to dissect differences in genetic profiles in limited samples. Nevertheless, large populations are needed to validate our results in the future. Besides, the incidence of HPV-related HNSCC is relatively lower in Taiwan than that in Western countries^38,39^. In our hospital, HPV status has been examined only in oropharyngeal SCC since 2012 and few samples had HPV information because of insufficient samples. As reported, the incidence rate is also low in our hospital (25%, 1/4)^38,39^. Second, because of the retrospective study design, there was a lack of adequate paraneoplastic tissue or blood samples to exclude germline mutations in our analysis. Third, although the findings related to the metabolic-associated genetic profiles, which could be predictive markers for PM in the future, were significant in short-term PMS HNSCC, it was still difficult to obtain pulmonary metastatic tissues from computed tomography-guided biopsy before this procedure. Since these mutations exist in metastatic lesions, future predictive biomarkers may be developed via liquid biopsy, and adjuvant target drugs after metastasectomy should be warranted.

In conclusion, the outcomes of PM were different according to the histological types of primary head and neck malignancies. For HNSCC, time to distant metastasis is a significantly predictive factor for the efficacy of PM. Metabolic-associated dysfunction, such as dysfunctions related to BCAA consumption, is the most important signaling pathway in disease progression and metastasis. Further validation is warranted.

## Material and Methods

### Study design and patient selection

This was a single-institute, retrospective, cohort study. From January 1, 2003 to December 31, 2012, a total of 59 patients with primary head and neck malignancies undergoing PM at Taipei Veterans General Hospital were enrolled. We screened HPV status by checking p16^INK4A^ expression, and tobacco status was also recorded in our cohort. The method used for p16^INK4A^ staining was described in our previous study^40^. After excluding patients whose primary cancer was not located in the head or neck, 49 patients with head and neck primary malignancies were selected, including 20 patients with HNSCC, 7 with NPC, 16 with TC, 3 with ACC, and 3 with other types of cancer (Table 1). The flowchart of the inclusion and exclusion criteria is presented in Fig. S1. To assess the effect of specific genes on survival outcome after PM, only matched pulmonary metastatic samples were selected for sequencing.

The study was reviewed and approved by the institutional review board of TPE-VGH (IRB No. 2016-01-011CC). Informed consent was obtained according to the guidelines of the institutional review board of TPE-VGH. All experiments involving human participants were also performed in accordance with relevant guidelines and regulations.

### Surgical condition

Patients who undergo PM should fit the indications as follows: pulmonary metastases appeared to be completely resectable based on preoperative imaging studies, the metastasectomy was deemed as technically feasible, the patient’s cardiopulmonary function was adequate to tolerate the operation, the primary tumor had been controlled, and extrapulmonary metastatic lesions were either absent or, if present, were controlled^41,42^. We selected patients whose pulmonary metastases were completely resected. The patients whose lesions could not be completely resected were not enrolled in the study.

### Clinical outcomes

Time to distant metastasis was calculated using the data of complete curative treatment, such as chemoradiotherapy, surgery, or adjuvant therapy, to the distant metastasis diagnosed from an imaging study. PMS was calculated from the date of PM to the date of death. OS was calculated from the date of disease diagnosed to the date of death or the last follow-up (Fig. 1a). To identify the patients with survival benefit after PM, patients were divided into short- and long-term groups according to PMS. The cutoff point for the short- and long-term groups was 24 months because the median survival after PM was 24 months in previous studies^9–11^.

### Tumor sample preparation and next-generation sequencing

Tumor specimens obtained at the time of PM were formalin fixed following standard protocols. The archived formalin-fixed, paraffin-embedded (FFPE) blocks were stored at an ambient temperature. The library was constructed by an Illumina Truseq Exome Library prep kit. Briefly, genomic DNA was fragmented to approximately 200 bp in length and ligated to indexing adapters to generate a sequencing library. The resulting library was then hybridized to biotin-labeled probes targeting exon regions. Finally, probes hybridized to the targeted regions were captured by streptavidin magnetic beads. Following several cycles of PCR, the library mixture was ready for quality check and quantification. After concentration adjustment, the library mixture was denatured and diluted for next-generation sequencing, which was performed via paired-end 150 cycle sequencing within the NextSeq 500 sequencer.

### Bioinformatics analysis

The FASTX-Toolkit (http://hannonlab.cshl.edu/fastx_toolkit/) was used to perform the sequencing quality processes. The sequence quality processing contained three steps. The first step was adapter trimming. The command “fastx_clipper” was used to do perform the adapter trimming. The second step was removing the poor-quality sequencing reads. The command was “fastq_quality_filter –Q33 –q 30 –p 70”. “-q 30” indicated that the minimum phred score was 30 (30 indicates that the sequencing error rate of a base is 0.01%) and “-p 70” indicates that the minimum percent of bases must have “-q” quality ≥70%. The third step was removing unpaired sequencing reads. Sequences were retained if both forward and reverse sequencing reads passed the first step.

Bowtie2 (http://bowtie.cbcb.umd.edu) was used as an efficient sequence alignment tool to align the obtained reads with the human genome (Grch38.p2)^43^. According to the results of the sequence alignment, the reads with only one chromosome location were retained for further analysis. Samtools (http://www.htslib.org/) was used to perform duplicate marking and editing of the format of the alignment results. GATK (https://software.broadinstitute.org/gatk/) was used to identify genetic variants according to the Samtools results.

The possible significant variants after sequencing were selected according to two criteria: meeting the read/depth selection and excluding variants with minor allele frequency (MAF) >1% in global or Taiwan BioBank database. Three read/depth selected criteria were used to identify these genetic variants as follows: variants with sequencing depth >20 and mutation frequency ≥5%, variants with sequencing depth >50 and mutation frequency ≥3%, or variants with sequencing depth >100 and mutation frequency ≥2%. If the variants fit one of the selected criteria, the variants would be enrolled in the groups for analysis. The read coverage was required to exceed 10, the genetic variants needed to be nonsynonymous, and the genetic variants were considered or predicted as pathogenic or possibly/probably functionally impaired. Several public domain databases were applied to annotate the identified genetic variants. These databases are described as follows. dbSNP (ftp://ftp.ncbi.nlm.nih.gov/snp) offered the frequency of genetic variants in different populations. Clinvar (ftp://ftp.ncbi.nlm.nih.gov/pub/clinvar) offered evidence-based genetic variants information about diseases. Cosmic (https://cancer.sanger.ac.uk/cosmic) offered the cancer-related information of the genetic variants. CADD (https://cadd.gs.washington.edu) offered the comprehensive prediction information of genetic variants.

After fulfilling these selection criteria, the significant genetic variants were further analyzed by using DAVID Bioinformatics Resources software version 6.7 (https://david.ncifcrf.gov/)^44^, IPA (QIAGEN Inc., https://www.qiagenbioinformatics.com/products/ingenuity-pathway-analysis)^45^, and STRING (https://string-db.org/)^46^. DAVID Bioinformatics Resources software version 6.7 provided a comprehensive set of functional annotation tools to understand biological meaning, such as KEGG pathway mapping and gene ontology (GO) enrichment analysis^44^. The networks were generated via the use of IPA^45^. And STRING, a database of known and predicted protein-protein interactions, also performed gene-set enrichment analysis, such as GO^46^.

First, DAVID and IPA were used to reveal signal transduction pathways and networks of the genes identifying from the signal pathways, respectively. Then, GO was analyzed by using DAVID and STRING individually, and comparing whether GO terms from these two databases were the same. The genes, which appeared in signal pathways and had the same GO terms, were selected as candidate genes. *In vitro* cellular functional experiments and immunohistochemistry (IHC) staining of tissue samples were perform to validate these candidate genes.

### SNP genotyping validation by Sanger sequencing

We performed PCR amplification product, including rs121912707 and rs80358294. Amplicons were subsequently subjected to Sanger sequencing. Information on the primers was described as below:

**Table:** 

SNP	Forward	Reverse	Product length
rs121912707	ATCCTCTGACCCCAAGTCC	CAACATCAGGCTAGCGAACA	218 bp
rs80358294	CTGGCTTGTGCTATGTTGGA	ACCCCAATCTTGACCATCAG	196 bp

### Immunohistochemistry (IHC) staining

Formalin-fixed paraffin-embedded tissue sections were obtained using a Leica Bond-MAX system (automated IHC staining systems). The sections were pretreated using heat-mediated antigen retrieval with sodium citrate buffer (pH 6, epitope retrieval solution 1) for 30 minutes. The section was then incubated with ALDH7A1 (HPA023296) (diluted 1/500) antibody for 60 minutes at room temperature and detected using an HRP-conjugated compact polymer system (Anti-Rabbit IgG–Poly-HRP) for 20 minutes at room temperature. The section was blocked with peroxide block for 5 minutes. DAB was used as the chromogen. The section was then counterstained with hematoxylin and mounted with DPX.

### Ki-67 staining

Formalin fixed paraffin embedded tissue sections were performed on a Leica Bond-MAX system(automated IHC staining systems). The section was pre-treated using heat mediated antigen retrieval with sodium EDTA buffer (pH9) for 30 min. The section was incubated with Ki-67 (diluted 1/50) for 60 mins at room temperature, then incubated with Second antibody (Rabbit anti-Mouse antibody) for 20 min at room temperature and detected using an HRP conjugated compact polymer system (Anti-Rabbit IgG–Poly-HRP) for 20 min at room temperature. The section was blocking with peroxide block for 5 minutes. DAB was used as the chromogen. The section was then counterstained with hematoxylin and mounted with DPX.

### Protein extraction and western blot

The control siRNA or cells were lysed with RIPA buffer (150 mM NaCl, 50 mM Tris-HCl pH 7.4, 0.25% Na deoxycholate, 5 mM EDTA, 1% Triton X-100, 5 mM EGTA, and 1% protease inhibitor cocktail) on ice for 30 minutes. The lysates were centrifuged at 14,000 rpm for 20 minutes. Equal amounts of protein samples were separated by SDS-PAGE and were then transferred onto polyvinylidene difluoride membranes (Millipore, Billerica, MA, USA). After blocking in 5% of BSA or milk, the membrane was incubated with anti-ALDH7A1 or actin antibodies. Blots were rinsed three to four times with PBS and incubated with horseradish peroxidase-conjugated secondary antibody. The blots were exposed to ECL Plus detection reagent. The images were analyzed by using Image QUANT TL8.1 (GE Healthcare, Marlborough, MA, US).

### Cell culture, transfection and cell viability assay

Cal27 and HSC3 cells were maintained in DMEM or EMEM, respectively, containing 10% fetal bovine serum, antibiotics (100 U/mL penicillin and 100 U/mL streptomycin), and 1% L-glutamine, at 37 °C in a humidified atmosphere with 5% CO_2_, and the culture medium was changed every two days. The cells were transfected with 40 nM of ALDH7A1 siRNA for 24 h followed by replacing the culture medium with fresh medium. The parental or transfected cells were then trypsinized and seeded into a 24-well culture dish at a density of 1 × 10^5^ cells/well for the indicated time periods (24 and 48 h); the viable cells were then evaluated by using the MTT assay method.

### Bromodeoxyuridine (BrdU) labeling and detection

The CAL27 or HSC3 cells were incubated with the BrdU labeling solution for 2 hours followed by fixing with 4% paraformaldehyde for 15 min at room temperature. The fixed cells were then permeabilized with 0.3% triton x-100 for 15 min followed by DNA hydrolysis with 0.2 M HCL solution for another 20 min. The cells were then added anti-BrdU antibody for 1 h at room temperature followed by incubating with anti-mouse FITC-conjugated antibody plus with DAPI solution. The fluorescence of the cells were then investigated under a leica microscopy.

### Ethidium bromide/Acridine orange stain

To analyze the effects of ALDH7A1 knockdown in cellular apoptosis, control siRNA or ALDH7A1 siRNA transfected CAL27 and HSC3 cells were washed with PBS twice followed by incubating with the 1 ml of Ethidium bromide/Acridine orange-containing PBS solution (Ethidium bromide: 3 ng/ml, Acridine orange: 5 ng/ml) for 5 min. The staining cells were then investigated under a leica microscopy. The living cells represented as green color, the apoptotic cells represented as orange color and the necrotic or late apoptotic cells represented as red color^47,48^.

### Statistical analyses

To identify the patients who experienced a survival benefit after PM, the independent factors for PMS longer than 24 months were analyzed with univariate and multivariate logistic regressions. The factors with values of *P* < 0.05 in the univariate analyses were included in the multivariate regressions. Survival was estimated using the Kaplan-Meier method and log-rank test. A two-sided value of *P* < 0.05 was regarded as statistically significant. Statistical analysis was performed using the SPSS software 18th version (SPSS Inc., Chicago, IL, USA).

## Supplementary information

Supplementary information

Supplemental Table S4-S12
