# Supplementary material for: Clinical, pathophysiologic, and genomic analysis of the outcomes of primary head and neck malignancy after pulmonary metastasectomy
Source: Sci Rep. 2019 Sep 9;9:12913. doi: 10.1038/s41598-019-49212-y (PMC6733860; doi:10.1038/s41598-019-49212-y)
Supplement: Supplementary file 1 — Supplementary information [file 41598_2019_49212_MOESM1_ESM.doc]

Clinical, pathophysiologic, and genomic analysis of the outcomes of primary head and neck malignancy after pulmonary metastasectomy

Hsueh-Ju Lu, MD^1,2,3^, Chih-Cheng Hsieh, MD^4,5^, Chi-Chun Yeh, MD^6^, Yi-Chen Yeh, MD^5,7^, Chun-Chi Wu, PhD^14,15^, Feng-Sheng Wang, PhD^8^, Jin-Mei Lai, PhD^9^, Muh-Hwa Yang, MD, PhD^10.11^, Cheng-Hsu Wang, MD^12^, Chi-Ying F. Huang, PhD^3,13*^, Peter Mu-Hsin Chang, MD, PhD^5,10*^

^1^Division of Medical Oncology, Department of Internal Medicine, Chung Shan Medical University Hospital, Taichung, Taiwan

^2^School of Medicine, Chung Shan Medical University, Taichung, Taiwan

^3^Program in Molecular Medicine, School of Life Sciences, National Yang Ming University, Taipei, Taiwan

^4^Division of Thoracic Surgery, Department of Surgery, Taipei Veterans General Hospital, Taipei, Taiwan

^5^Faculty of Medicine, National Yang Ming University, Taipei, Taiwan

^6^Jin An Clinic, New Taipei City, Taiwan

^7^Department of Pathology and Laboratory Medicine, Taipei Veterans General Hospital, Taipei, Taiwan

^8^Department of Chemical Engineering, National Chung Cheng University, Chiayi, Taiwan

^9^Department of Life Science, Fu Jen Catholic University, New Taipei City, Taiwan

^10^Division of Medical Oncology, Department of Oncology, Taipei Veterans General Hospital, Taipei, Taiwan

^11^Institute of Clinical Medicine, National Yang Ming University, Taipei, Taiwan

^12^Cancer Center, Keelung Chang Gang Memorial Hospital, Keelung, Taiwan

^13^Institute of Biopharmaceutical Sciences, National Yang Ming University, Taiwan

^14^Institute of Medicine, Chung Shan Medical University, Taichung, Taiwan
^15^Department of Medical Research, Chung Shan Medical University Hospital, 
Taichung, Taiwan

**Disclosure**: The authors have no conflicts of interest to declare.

***Corresponding authors**: Peter Mu-Hsin Chang, Division of Medical Oncology, Department of Oncology, Taipei Veterans General Hospital, No. 201, Sec. 2, Shih-Pai Road, Taipei 112, Taiwan. E-mail: [ptchang@vghtpe.gov.tw](mailto:ptchang@vghtpe.gov.tw); and

Chi-Ying F. Huang, Institute of Biopharmaceutical Sciences, National Yang-Ming University, No. 155, Section 2, Linong Street, Taipei 112, Taiwan Taipei 112, Taiwan. E-mail: [cyhuang5@ym.edu.tw](mailto:cyhuang5@ym.edu.tw)

**Supplementary Figures and Tables**

**Figure legends**

**Figure S1.** **Inclusion and exclusion criteria.** A total of 49 patients with head and neck primary malignancy underwent pulmonary metastasectomy at Taipei Veterans General Hospital; the sample included 20 patients with HNSCC, 7 with NPC, 16 with TC, 3 with ACC, and 3 with other types of cancer. After analyzing clinical presentations, only 20 HNSCC patients were evaluated for whole-exome sequencing. ACC, adenoid cystic carcinoma; HNSCC, head and neck squamous cell carcinoma; NPC, nasopharyngeal carcinoma; TC, thyroid carcinoma.

**Figure S2.** **Median PMS and OS of 20 HNSCC patients.** (a) The 20 patients with HNSCC were classified into short- and long-term PMS groups according to PMS (<24 and >24 months, respectively). The median PMS and OS between these two groups were 13.7 vs. 84.2 months (*P* < 0.001) and 23.9 vs. 108.8 months (*P* < 0.001), respectively. (b) After excluding unqualified HNSCC samples, only six pulmonary metastatic samples were sequenced. The median PMS and OS of these matched patients in the two groups were 11.8 vs. 42.7 months (*P* = 0.025) and 23.9 vs. 89.8 months (*P* = 0.025), respectively. HNSCC, head and neck squamous cell carcinoma; OS, overall survival; PMS, postmetastasectomy survival.

**Figure S3. Sanger sequencing for FZD4.** Sanger sequencing showed that the point mutation (*FZD4*, missense mutation, c.1250G>C, rs80358294) was compatible with the results from the high-throughput sequencing (WES).

**Figure S4. The Western blot of adding siALDH7A1 for CAL-27 and HSC-3 cells.** The CAL-27 and HSC-3 cells were either transfected with vehicle or ALDH7A1 siRNA for indicated time as described in Figure 5c, the cells were then harvested, lyzed and subjected to the western blot using anti-ALDH7A1 antibody.

**Figure S5. The genetic profiles between HNSCC, ACC, and other salivary gland cancers.** In the Morris et al. study, 151 recurrent metastatic head and neck cancer patients were enrolled in a Memorial Sloan Kettering Cancer Center cohort.^1^ Among these patients, a total of 86 patients with distant metastatic lesions were selected for a comparison of genetic profiles; the sample included 36 patients with HNSCC, 35 with ACC, and 15 with other salivary gland carcinomas. As shown in the figure, the genetic profiles between ACC and HNSCC patients were more similar than those between the patients with ACC and other salivary gland malignancies.

**Reference**

1 Morris, L. G. *et al.* The Molecular Landscape of Recurrent and Metastatic Head and Neck Cancers: Insights From a Precision Oncology Sequencing Platform. *JAMA oncology*, doi:10.1001/jamaoncol.2016.1790 (2016).


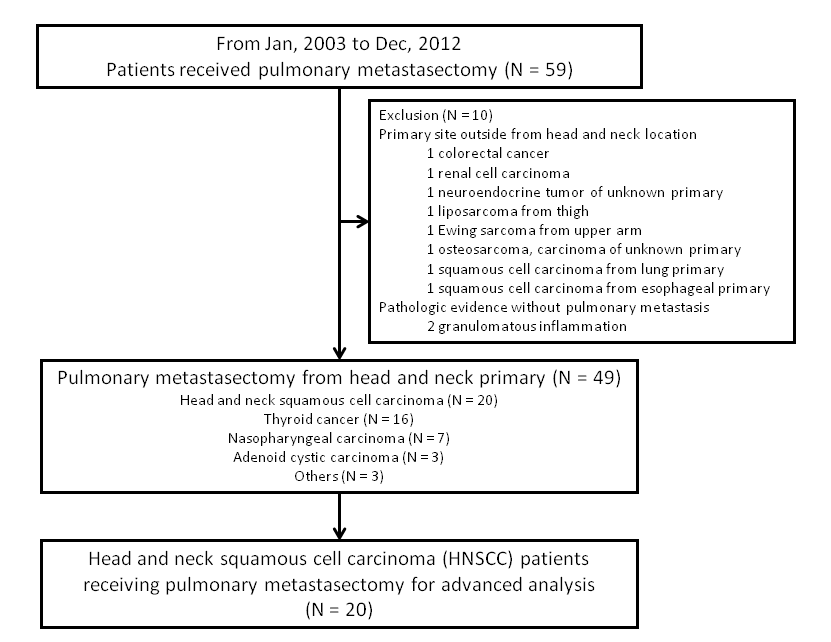
**Figure S1.** **Inclusion and exclusion criteria.**

**Figure S2.** **Median PMS and OS of 20 HNSCC patients.**

**
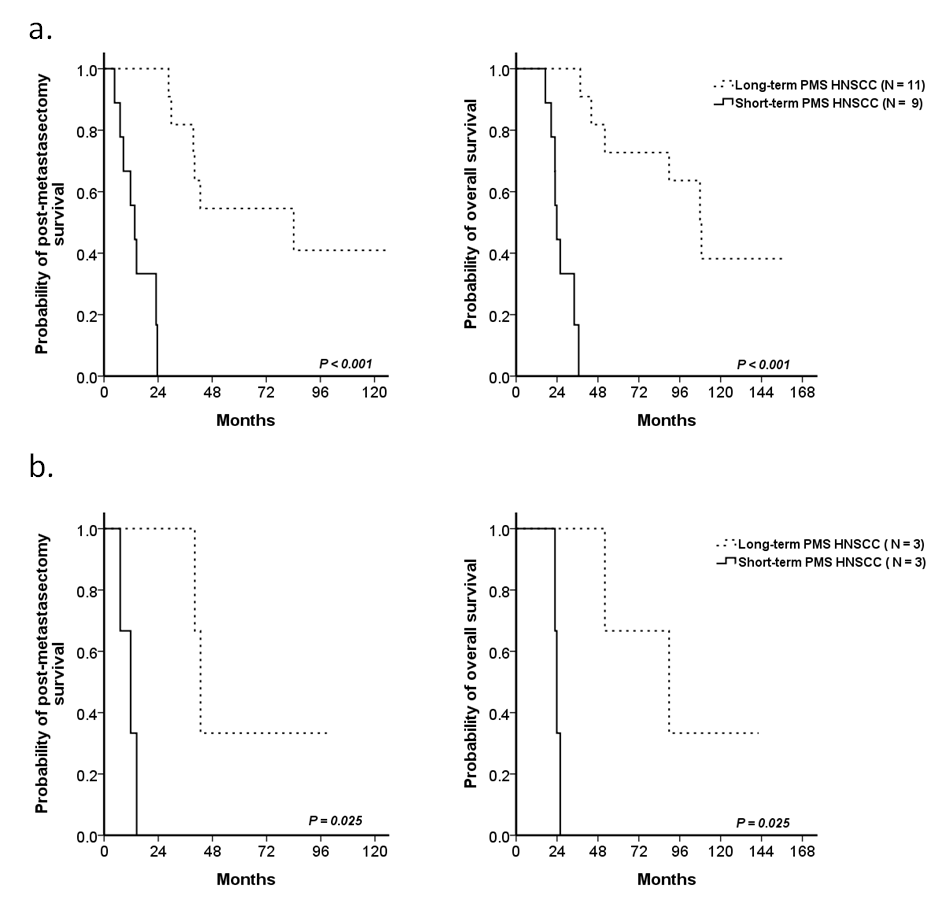
**

**Figure S3. Sanger sequencing for FZD4.**

**
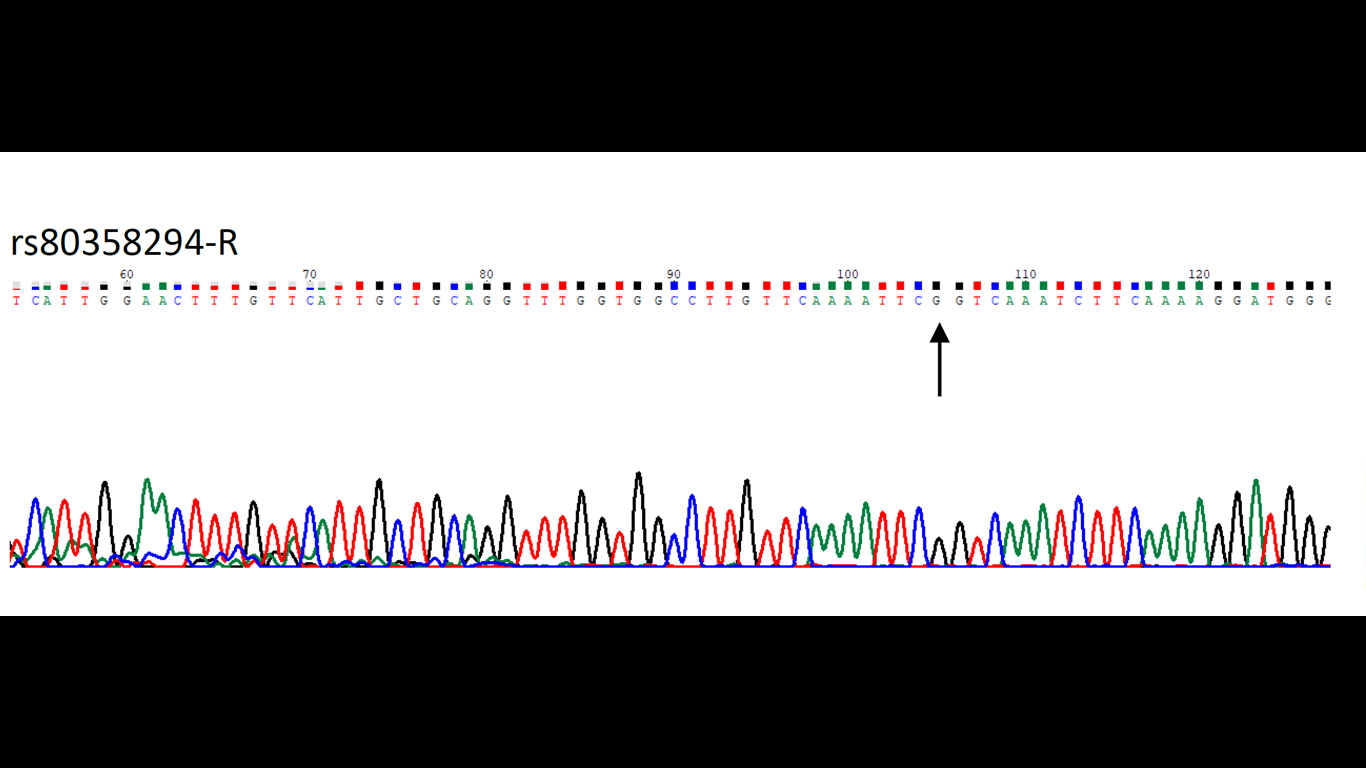
**

**Figure S4. The Western blot of ALDH7A1 from both CAL-27 and HSC-3 cells.**

**
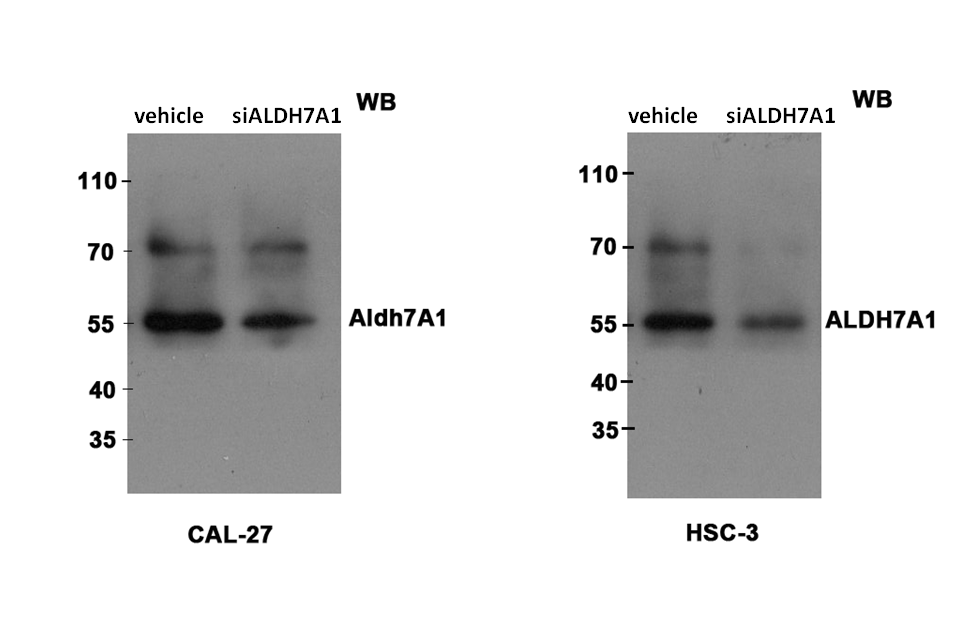
**


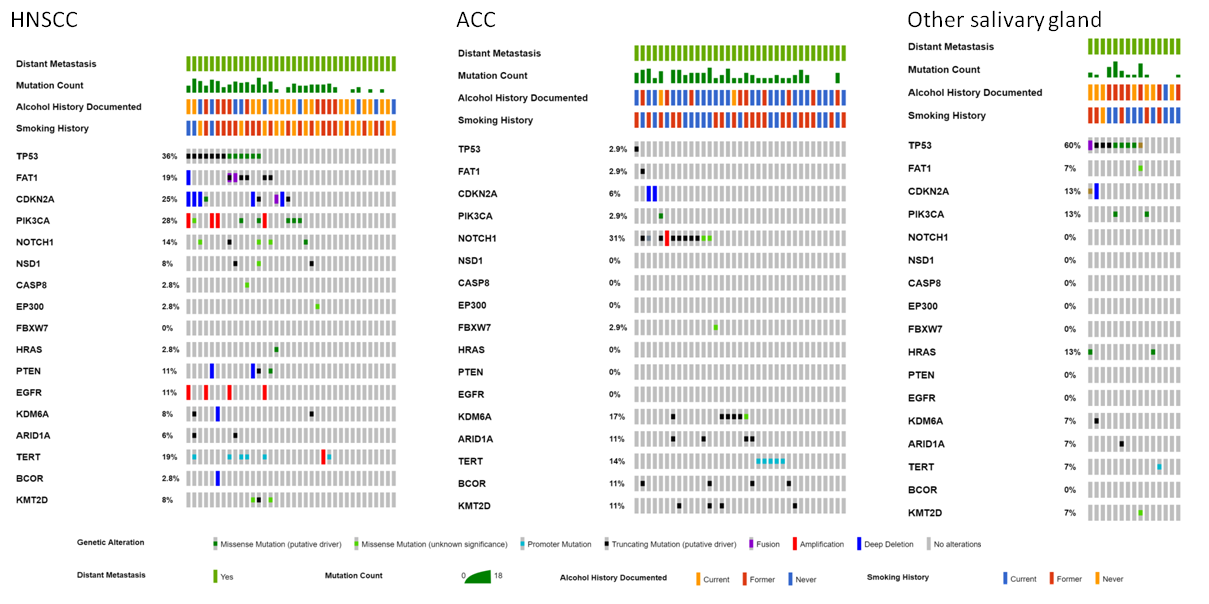
**Figure S5. The genetic profiles between HNSCC, ACC, and other salivary gland cancers.**

Table S1. Univariate and multivariate Logistic regression analysis for the independent factors of the efficacy of PM

| Characteristic | Univariate | Multivariate | |
| --- | --- | --- | --- |
|  | *P* value | *P* value | OR(95% CI) |
| Age > 60 (years) | 0.783 |  |  |
| Primary tumor location  Oral cavity vs. non-oral cavity | 0.888 |  |  |
| Initial clinical staging  T staging > 3  N staging positive | 0.619  0.935 |  |  |
| Curative surgery | 0.045 | 0.116 | 1.673(-0.125-1.014) |
| Pathologic feature after curative surgery  Extracapsular spread  Margin < 0.5cm  Lymphovascular invasion  Perineural invasion  p16^INK4A^ | 0.711  0.910  0.235  0.599  0.423 |  |  |
| Time to distal metastasis after curative treatment < 12 months | 0.010 | 0.025 | - 2.500[-1.032 - (-0.079)] |
| Pulmonary metastatic number > 2 | 0.605 |  |  |
| Bilateral pulmonary metastases | 0.518 |  |  |
| Pathologic feature of matched pulmonary metastatic sample  Tumor size > 2cm  Margin < 0.5cm  Lymphvascular invasion  Mediastinum organ involvement | 0.369  0.621  0.783  0.440 |  |  |
| Smoking | 0.913 |  |  |

Abbreviation: OR, odds ratio; CI, confidence interval

Table S2. Basic characteristics of patients receiving pulmonary metastasectomy

|  | Age, | Gender | Primary tumor site | Histological type | Initial pathologic staging | Brief history | Treatment after pulmonary metastasectomy | OS | PMS | Death |
| --- | --- | --- | --- | --- | --- | --- | --- | --- | --- | --- |
| Case 1 | 86.0 | Male | hypopharynx | SCC | pT4aN2b | Definitive wide excision plus LN dissection followed up adjuvant radiotherapy, complete remission; one metastatic nodule approximately 3.0 x 2.2 x 2.2 cm at RLL was resected 8.7 months after diagnosis | Platinum-based palliative chemotherapy | 23.9 | 14.4 | Yes |
| Case 2 | 60.9 | Male | oropharynx | SCC | pT4aN1 | Definitive wide excision plus LN dissection followed up adjuvant chemoradiotherapy; two metastatic nodules were found at RUL and LUL 13.5 months after diagnosis. LUL lesion approximately 2.3 x 1.8 x 1.6 cm was resected. | Platinum-based palliative chemotherapy | 25.9 | 11.8 | Yes |
| Case 3 | 47.1 | Male | oropharynx | SCC | pT4aN2b | Induction chemotherapy with follow-up definitive wide excision plus LN dissection and adjuvant chemoradiotherapy; one LUL nodule and mediastinal LN metastases were identified 14.7 months after diagnosis. Resected LUL nodule was about 2.3 x 0.4 x 0.4 cm. | Platinum-based palliative chemotherapy plus local radiation therapy for bone metastasis | 22.8 | 7.1 | Yes |
| Case 4 | 38.9 | Male | hypopharynx | SCC | pT2N2b | Definitive wide excision plus LN dissection with follow-up adjuvant radiotherapy; multiple bilateral lung metastases 11.6 months after diagnosis, sequential wedge resection for left and right side lesions | Cetuximab plus platinum-based palliative chemotherapy | 52.1 | 40.2 | Yes |
| Case 5 | 51.3 | Male | hypopharynx | SCC | pT3N2b | Definitive wide excision plus LN dissection with follow-up adjuvant chemoradiotherapy, three metastatic lesions at RUL/RML/LLL 25.3 months after diagnosis, radiofrequency ablation and wedge resection were administered several times | Platinum-based palliative chemotherapy | 89.8 | 42.7 | Yes |
| Case 6 | 54.4 | Male | hypopharynx | SCC | pT4aN2b | Definitive wide excision plus LN dissection with follow-up adjuvant chemoradiotherapy; two metastatic nodule lesions at bilateral lung apices 42.4 months after diagnosis, sequential wedge resections for RUL and LUL tumors (approximately 1.2 x 0.7 x 0.7 cm and 1.3 x 1.0 x 1.0 cm, respectively) | Platinum-base palliative chemotherapy | 142.3 | 98.7 | No |
| Case 7 | 39.6 | Female | oral cavity | ACC | pT4aN0 | Definitive wide excision plus LN dissection with follow-up adjuvant chemoradiotherapy; two metastatic nodules at RUL and RML were resected (1.4 x 1.0 x 1.2 cm and 1.2 x 0.9 x 0.6 cm, respectively) 41.8 months after diagnosis. | Cetuximab plus platinum-based palliative chemotherapy, metronomic therapy, and MTX | 129.9 | 86.8 | No |
| Case 8 | 46.2 | Female | right maxillary sinus and nasal cavity | ACC | Unknown | Definitive wide excision plus adjuvant radiotherapy; two metastatic nodules at RUL and RML (1.2 x 0.7 x 0.7 cm and 1.3 x 0.8 x 0.5 cm, respectively) were resected 108.7 months after diagnosis. | Loss regular follow-up | 195.0 | 79.9 | No* |

Table S3. Selection criteria for pathogenic variants

|  | Short-term PMS HNSCC | Long-term PMS HNSCC | ACC |
| --- | --- | --- | --- |
| Raw data | 8566 variants | 8083 variants | 8572 variants |
| Read and Depth selection | 2412 variants | 2323 variants | 1816 variants |
| Exclude MAF > 1% in global or Taiwan BioBank database* | 2301 variants  (1036 genes) | 2201 variants  (1026 genes) | 2316 variants  (1060 genes) |

*Because only 15.5% (835/5386) of variants were annotated in MAF from global or Taiwan BioBank database, the variants without MAF evidence were all enrolled.
